# Poo Manager: Co‐Designing a Serious Computer Game to Improve Constipation Management Awareness in Carers of People With Intellectual Disabilities

**DOI:** 10.1049/htl2.70024

**Published:** 2025-11-03

**Authors:** Serena Daniel, Ruth Bishop, Stephen Howard, Sarah Lennard, Samantha Pye, Richard Laugharne, Christopher James, Rohit Shankar

**Affiliations:** ^1^ University of Exeter Medical School Truro UK; ^2^ Cornwall Intellectual Disability Equitable Research (CIDER) Cornwall Partnership NHS Foundation Trust Truro UK; ^3^ Games Design, Cornwall College Camborne UK; ^4^ CIDER Peninsula School of Medicine University of Plymouth Plymouth UK; ^5^ Biomedical Engineering Institute University of Warwick Warwick UK

**Keywords:** biomedical communication, biomedical education, computer based training, health care, medical disorders

## Abstract

Approximately 2% of the population are people with intellectual disability (PwID). PwID are prone to multimorbidity, polypharmacy and premature mortality. Constipation is a prevalent and life‐threatening issue for PwID fostered by iatrogenic harm, ignorance and stigma in family, and health and care settings. Despite its clinical significance, educational tools targeting caregivers’ knowledge of constipation prevention and management in PwID are scarce. Serious games (SGs) offer a novel and engaging platform to educate carers and reduce health stigma. *Poo manager* was collaboratively designed by a team of clinicians, academics, game developers and people with lived experience. The game involves navigating a care home, identifying constipation risk factors and selecting appropriate management strategies through evidence‐based gameplay. A co‐production workshop involving PwID, their carers and clinicians provided feedback on gameplay. Evaluation was conducted using validated measures to examine intervention acceptability, appropriateness and adoption feasibility. Over 60% found the SG enjoyable and believed it could improve constipation management knowledge. Scores demonstrated high acceptability, appropriateness and feasibility, though some concerns around game usability were noted. *Poo manager* is a promising SG intervention with strong potential to enhance constipation care for PwID. Further development and formal effectiveness evaluation are warranted to ensure impact on clinical outcomes.

## Graphical abstract

Many people with intellectual disability (PwID) are prone to life threatening constipation due to carer ignorance and stigma. Poo manager is a serious computer game to teach constipation care to carers of PwID. Co‐production of Poo Manager involved PwID, carers, clinicians and game design experts. The game uses humour and interactive play to reduce stigma, raise awareness and teach care. Most users found the game acceptable, enjoyable, appropriate and feasible to use.

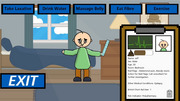

## 1 Introduction

### 1.1 Serious Games

Across the world, public health bodies and medical teams are increasingly turning to digital health interventions to diagnose, assess, treat and prevent a wide variety of health conditions [1, 2, 3]. At the forefront of this wave of inquiry are serious games (SGs) [4, 5]. SGs are games designed to have a non‐recreational purpose [6]. The gameplay performs an additional function ranging from teaching players a new skill [7, 8] to reducing stigma about a health condition [9, 10]. SGs can also engage users in specific behaviours that have the potential to improve their well‐being [11, 12, 13]. SGs have been particularly successful in the field of health education [14, 15]. SGs have successfully improved health professional/student knowledge in fields such as geriatrics [16], insulin therapy [17], cardiac life support [18] and major incident triage [19].

### 1.2 Intellectual Disability

People with intellectual disability (PwID) make up almost 2% of the UK population [20, 21]. Intellectual Disability can be defined as when a person has major difficulty or delay in acquiring skills across most developmental areas, which limits the ability of an affected person to learn and function at societally expected levels [22]. PwID are often heavily reliant on both family and professional carers, and many require lifelong support [23, 24, 25]. PwID die on average 20 years earlier than the rest of England's population [26]. They experience far higher rates of concurrent chronic health conditions such as epilepsy, ADHD, autism and gastro‐intestinal conditions [27].

### 1.3 Constipation

Constipation is defined by bowel symptoms (difficult or infrequent passage of stool, hardness of stool, or a feeling of incomplete evacuation) that may occur either in isolation or secondary to another underlying disorder [28].

It can be defined in clinical practice as opening bowels less than three times per week or using laxatives three or more times weekly [29]. Constipation is caused by a complex web of factors ranging from inadequate dietary fibre intake, reduced mobility, medical conditions, medication side effects and reduced fluid intake. Managing constipation includes avoiding such contributory factors and the use of laxatives [30]. If ignored, constipation can progress to faecal impaction, rectal prolapse, bowel obstruction and death [31].

### 1.4 Constipation and PwID

Constipation is a major health issue for PwID and is a significant cause of both mortality and morbidity in this community [26, 29, 32, 33, 34, 35]. It is a leading cause of emergency department admissions for this population [33]. In 2017, a systematic review examined the global prevalence of constipation as a comorbidity in PwID. The review included only English‐language studies published between 1990 and early 2016, resulting in the selection of 31 studies. Findings revealed that constipation was reported as a registered health problem in 30% to 70% of PwID across most studies. In some populations, prevalence rates were reported to exceed 95% [36].

Risk factors for constipation include PwID who are on medications with a high cholinergic burden, those who are immobile and those who are non‐verbal or struggle to communicate their needs [32, 33, 37]. There is also a correlation between severity of intellectual disability and risk of constipation [29].

Constipation is not only prevalent in this population, but also life‐threatening [34]. The most recent Learning Disability Mortality Review (LeDeR), published in 2024, reviewed 97 premature deaths of PwID across the UK throughout 2021 [26]. Approximately 23% (n = 22) of PwID who were identified as dying prematurely in the review had constipation listed as a long term health problem, and 13% (n = 6) had a constipation linked condition as the primary cause of death [26, 38]. Almost 40% (n = 39) were prescribed at least one laxative at their time of death [26]. Constipation prevention and management is therefore at the forefront of healthcare for PwID, particularly in England [39].

Despite recent England's National Health Service (NHS) efforts such as the ‘Constipation Campaign Took Kit’ [40], there are still many barriers to successful constipation prevention and management in PwID. Identified barriers include the stigma parents face when discussing bowel movements with health professionals, lack of knowledge of risk factors amongst family and carers and diagnostic overshadowing [41, 42, 43]. These barriers are particularly concerning given the reliance of PwID on the care of family and health professionals [23, 25, 34]. Iatrogenic harm is also a common issue in this population. Particularly as a large proportion of medications prescribed to this group have constipation as a side effect, especially those with a high anticholinergic burden [41]. To reduce the significant burden of constipation, PwID need holistic/person‐centred care that supports carers to identify and manage constipation at home [39, 41, 42].

### 1.5 Serious Games and Constipation in PwID

Increasing the knowledge and awareness among those who care for PwID is needed to improve the management of constipation in the community [42, 43]. Using an SG designed to educate health and social care staff could be an effective method of reducing the prevalence and impact of constipation in PwID [28]. Prior research in this field has concluded that no such game targeting the management, prevention and stigma of constipation exists, particularly for PwID [44].

## 2 Related Work

Despite their increased health burden, there have been very few healthcare SGs developed that target PwID and/or their carers. Most existing games that target PwID aim to improve their social, cognitive or practical skills [8, 45, 46]. A systematic review focused on the use of serious computer games to improve skills in PwID and/or autism spectrum disorder (ASD) found that these games are often used to address social and communication skills in individuals with ASD [22]. Some studies showed improvements in social behaviour, attention and emotion identification, results were inconsistent [45]. The scope of this review was also limited to SGs that educated PwID or ASD. It did not explore SGs which targeted linked outcomes such as reducing the stigma associated with a diagnosis or preventing or managing physical health conditions in this population. This is a common omission in the field [8, 47, 48, 49, 50] that further highlights how few SGs exist for this population which target medical outcomes, such as constipation, despite the significant comorbidities PwID suffer from [27].

Co‐production during development is vital to the success of any healthcare technology, including SGs, because it promotes acceptance by the target population [51]. Co‐production is an approach in which researchers, practitioners and those with lived experience work together, sharing power and responsibility [52]. Previous work by Tskinas et al. has suggested that PwID and those who assist and work with them must be included in the development of any serious game aimed at improving the lives of this demographic if it is to be successful [22, 47, 53]. This will inform the SG design process.

### 2.1 Aim

To develop an SG designed to improve awareness, teach management strategies and reduce the stigma of constipation targeted at those who care for PwID. The secondary aim was to investigate the initial views of end users of the game and readiness for implementation to inform its further development.

## 3 Application Design

The game was developed by lecturers and students on the Games Design for Industry course at Cornwall College in Cambourne in conjunction with the Cornwall Intellectual Disabilities and Research Team (CIDER) at the University of Plymouth medical school and the local NHS Trust [54].

The properties and design components of an SG are vital determinants of its effectiveness [55, 56, 57]. Therefore, the team drew on their previous experience in developing SG for healthcare to ensure successful game design, as well as from the results of a previous literature review on SGs for bowel movements [44].

Health, social and professional care staff (the intended audience) and PwID (experts with experience) were consulted throughout game development. This co‐production included discussions with carers about the game's care home setting and discussions with psychiatrists about the causes of constipation in this population.

### 3.1 Gameplay

The team developed a multi‐level single player platform game set in a care home for people with a range of intellectual disabilities. Example game play can be found at this link: https://youtu.be/HBFefjlIuWw?si=ITPk8wwRqlOsPgwf. Further details and numbered images of the gameplay itself are provided in supplementary information images 1–15.

**FIGURE 1:**
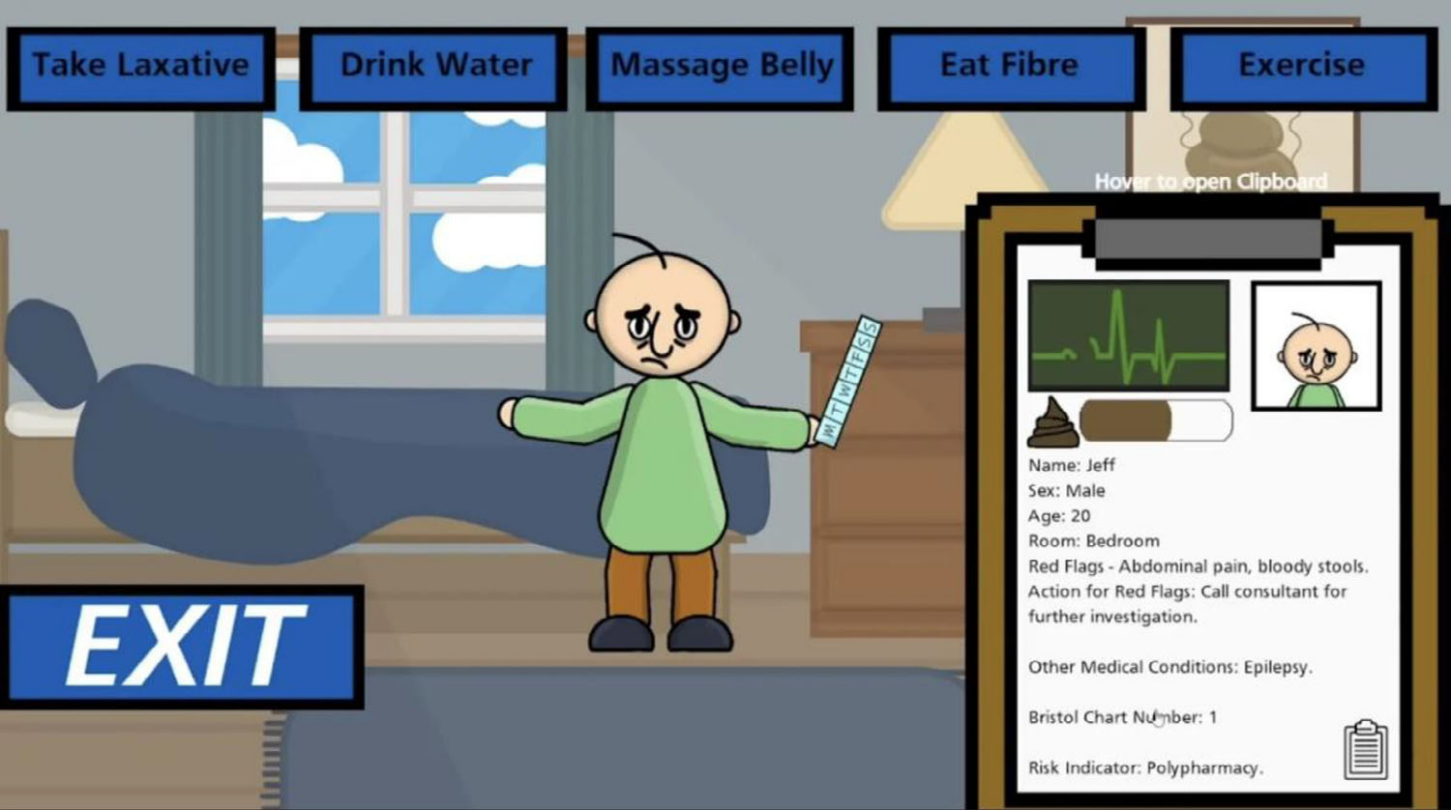
A screen shot of gameplay showing the player engaging with a patient and reading the vignette. The five boxes on the top of the screen are the pre‐determined actions or treatment options the player can choose from. The clip board represents the vignette and contains the poo meter in brown and the health meter in green.

The player is represented by an avatar who roams a multi‐room care establishment, meeting people with disabilities who are suffering from constipation (Figure 1). The player engages with each person and treats the constipation by choosing from a range of pre‐selected treatments. The aim is to prevent the patient's ‘poop meter’ becoming full and killing the person.

**FIGURE 2:**
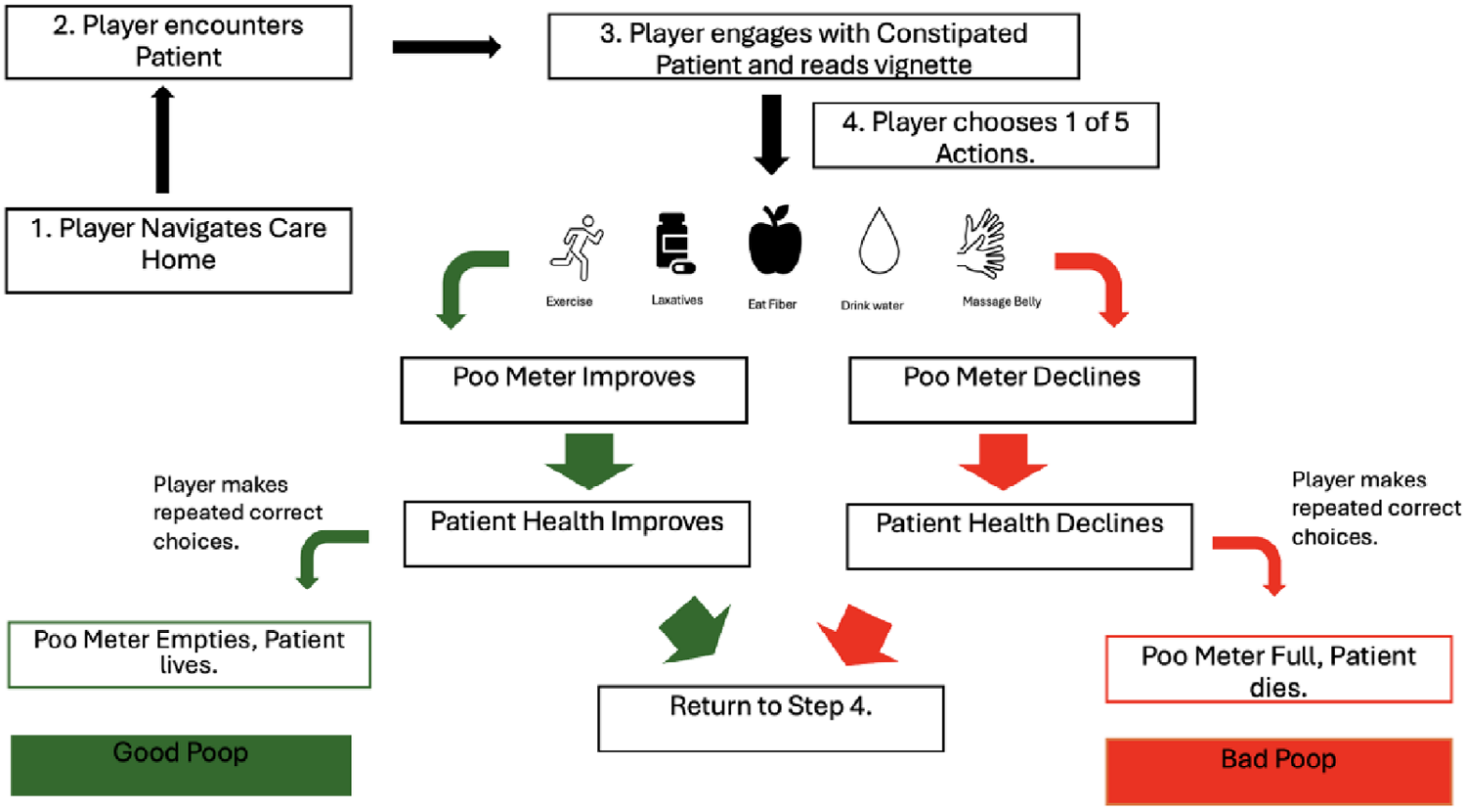
A flow chart detailing the steps of gameplay. Step 1: begins with the patient navigating the care home. Step 2: The player encounters the patient. Step 3: The player engages with constipated patient and reads vignette. Then depending on if the patient chooses the correct or incorrect answer (randomised) the poo meter either improve or declines. They then return to Step 4 and choose again (randomised). This repeats until the poo meter is full and the Patient recovers and has a ‘good poop’, or until they pass away and do a ‘bad poop’.

Once the player enters a room, they are presented with a person, treatment options and their health indicator. Players must read the vignette of information displayed on a board beside each person and select a treatment from a set of pre‐selected evidence based clinical actions. If the player selects the correct treatment option, the person's health improves and their ‘poo meter’ value decreases. If this is repeated, eventually the person goes to the bathroom for a ‘good poop’. If the wrong option is selected, the value on the person's ‘poop meter’ increases. The ‘poop meter’ represents the severity of their constipation. If wrong actions are repeated, the ‘poop meter’ becomes full, and their health declines. Then the person goes to the bathroom for a ‘bad poop’ and dies (Figure 2). This is represented by a red flatline on the health indicator. The success or failure of these steps will teach players how to manage constipation via procedural learning [58].

### 3.2 Game Design Choices

The treatment options are randomised so that no option is correct twice in a row. This teaches the audience that there is no one correct option to treat constipation and that plans must be varied and individualised.

The game was designed to be played on a PC to enable easy distribution amongst care providers. The player navigates the map using the ‘WASD’ keys on a PC keyboard, each representing up, left, down and right respectively. Patient vignettes and information can be read by holding the mouse over the clipboard icon. Actions are selected using the PC mouse right click.

The player navigates through the map in a 2D fashion, encountering rooms where the people reside. The map itself and the people's rooms are colourful and cartoonish. Good poops and bad poops are represented by humorous animations designed to keep the learning light and fun. The aim is to reduce the stigma associated with discussing bowel movements amongst clinical staff and professional carers through both humour and understanding [59].

The five treatment actions offered to the player were ‘take laxative’, ‘drink water’, ‘massage belly’, ‘eat fibre’ and ‘exercise’. These closely mimic the management options for constipation in adults provided in the NICE guidelines [30].

The patient vignette on the clipboard included their name, sex, age, room, comorbidities, Bristol Stool Chart Number, risk indicator and reg flags identified for constipation. This encourages the player to consider each patient as an individual, teaches them to look for constipation risk factors and improves their Bristol Stool Chart Literacy. Recognition of these features will be encouraged by progression through the game.

The difficulty of each level increases gradually. In the first level the player learns the basics of constipation management and how to treat one person. Further levels increase in complexity in terms of the number of people, the area the character patrols and eventually the number of care homes. This challenging gameplay is vital for maintaining player motivation [55].

## 4 User‐Experience Evaluation

A co‐production day in May 2024 was organised where attendees were invited to evaluate the game and guide improvements to its design. Individuals were identified through professional networks and local advertising. This included PwID as peer researchers, carers, specialist nurses, psychiatrists and occupational therapists working in both health and social care settings. During the co‐production day, the game developers worked alongside attendees to explain and play the game. Working in this way, immediate feedback could be given. This was formalised with the use of a survey to ensure all views and experiences were captured.

### 4.1 Survey Development

A survey was developed (supplementary information 16) that was divided into 4 sections: ‘about you’, ‘constipation management’, ‘the game poo manager’ and acceptability of intervention measure (AIM), intervention appropriateness measure (IAM) and feasibility of intervention measure (FIM). It aimed to gather basic demographic information, views on the importance of constipation management and views on the usefulness and enjoyment of the game. An option to provide a free text response was given for additional thoughts or comments.

The final section of the survey focused on questions relating to the implementation outcomes of acceptability, appropriateness and feasibility to determine early indicators of implementation success. The questions were adapted from the AIM, IAM and FIM, which were developed as psychometrically validated tools to evaluate implementation outcomes. These measures specifically assess whether an intervention is perceived as acceptable, appropriate and feasible [60]. Specifically, if the intervention is viewed as feasible, acceptable and appropriate, the measures have been developed so they can be adapted to investigate interventions in a variety of settings and completed by different stakeholders [61]. Within each measure the respondents were asked to rate how strongly they agreed with four statements using a Likert scale. Staff members were on hand to assist panel members, particularly those with PwID. There was also an option to conduct a video interview instead.

### 4.2 Data Analysis

Most survey items were summarised using the percentage response rate to each question. For primary outcomes, AIM, IAM, and FIM were calculated by summing the responses for the individual questions. Each scale consists of 4 items, with each item scored from 1 to 5; the scale range is restricted from 5 to 20. Free text comments were summarised.

## 5 Results of the User‐Experience Evaluation

### 5.1 Demographics and Response Rate

The survey had 24 respondents, 10 of whom identified as male and 14 as female. A wide range of ages were included, with over half of responders falling into the 31–50 age groups (13/24). Not all the 24 respondents completed all questions of the survey. Twenty‐three responded to the demographics, attitudes to constipation and poo manager section. Nineteen responded to the final AIM, FIM and IAM section.

Of the respondents, just over a third (n = 8, 30%) had an intellectual disability, over two‐thirds (n = 16, 70%) supported a PwID as part of their work, and a quarter (n = 6, 26%) had a family member with an intellectual disability.

### 5.2 Attitudes to Constipation Management

Over half of respondents (n = 13, 57%) had experienced constipation personally, and nearly half (n = 10, 43%) had supported someone else with the condition.

All participants (n = 23, 100%) agreed that training in constipation management was either ‘important’ or ‘very important’. Attitudes towards the role of medication in constipation management were more varied. Over half (n = 13, 57%) considered medication ‘important’ or ‘very important’, while the rest (n = 10, 43%) rated it as neutral.

In contrast, all respondents (n = 23, 100%) believed that non‐pharmaceutical methods were “important or very important” for managing constipation. Additionally, all participants (n = 23, 100%) considered understanding the potential harms of constipation to be ‘important’ or ‘very important’.

### 5.3 Attitudes to Poo Manager

Approximately 61% (n = 14) of respondents either ‘agreed’ or ‘strongly agreed’ that the game was user‐friendly, while 22% (n = 5) ‘disagreed’ or ‘strongly disagreed’ with this assertion.

Despite these concerns, nearly two‐thirds (n = 15, 65%) found the game enjoyable to play, whereas 39% (n = 9) either ‘disagreed’ or ‘were neutral’.

Similarly, nearly two‐thirds (n = 14, 64%) of participants believed that the game would be beneficial in helping care givers learn about managing constipation, with just over a third (n = 8, 36%) either disagreeing or remaining neutral. When asked if the poo manager would assist care givers in learning alternative methods for preventing and treating constipation, 36% (n = 8) strongly agreed, 55% agreed (n = 12) and 9% (n = 2) disagreed.

Responses regarding the potential uptake of the game were more varied, as 41% (n = 9) of respondents believed that care givers and care organisations would adopt the game, 45% (n = 10) neither agreeing nor disagreeing and 14% (n = 3) disagreeing. Over half of the participants (n = 13, 57%) felt that the game would be effective in reducing constipation, while 40% (n = 9) neither agreed nor disagreed and 4% (n = 1) disagreed with this claim.

### 5.4 Implementation Outcomes (Acceptability, Appropriateness and Feasibility)

**FIGURE 3:**
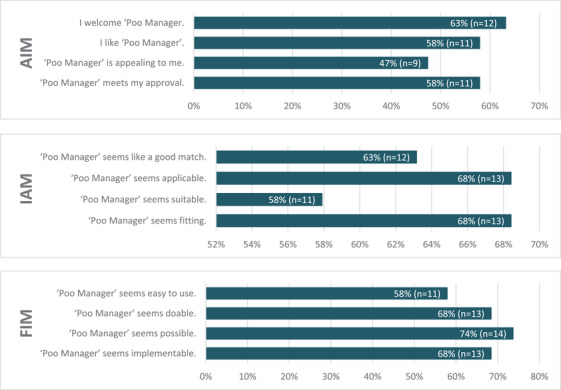
Proportion of respondents who agreed/completely agreed with survey questions about the acceptability, appropriateness and feasibility of poo manager. AIM, acceptability of intervention measure; IAM, intervention appropriateness measure; and FIM, feasibility of intervention measure.

Figure 3 represents the proportions of respondents who agreed or completely agreed with the implementation measures used (AIM, IAM and FIM). These assessed their views on the acceptability, appropriateness and feasibility of implementing poo manager as a tool to improve management of constipation, as previously discussed.

The ‘poo manager’ intervention was well received by participants, as reflected in the AIM. Across all four items assessed, the majority of participants expressed their agreement or strong agreement. Specifically, 11 participants agreed or completely agreed that the intervention met their ‘approval’, while 9 agreed or completely agreed that it was appealing and 12 agreed or completely agreed that they ‘welcomed’ it. 11 participants agreed or completely agreed that they ‘liked’ the intervention. Disagreement levels were relatively low, with between 2 and 5 participants disagreeing or strongly disagreeing with the positive statements across all four AIM items.

Perceptions of the appropriateness of the intervention were similarly positive (IAM). In the IAM, the majority of respondents agreed or strongly agreed that ‘poo manager’ was ‘fitting’ (n = 13), ‘suitable’ (n = 11), ‘applicable’ (n = 13) and a ‘good match’ (n = 12) for the intended context. Only one participant disagreed with each item, and no participants selected ‘completely disagree’ for any IAM item. This suggests a broad consensus on the intervention's appropriateness.

Findings from the FIM indicated that most participants viewed ‘poo manager’ as a feasible intervention. Fourteen participants agreed or completely agreed that it was possible to implement, while 13 agreed or completely agreed it was implementable and doable, respectively. Eleven participants agreed or completely agreed it was easy to use. A small number of respondents expressed disagreement, particularly regarding the intervention's ease of use (n = 4), though the majority of the FIM responses were still positive (n = 51/76).

### 5.5 Free Text Reflections of Respondents to Poo Manager

11 free‐text responses were recorded during the survey. These highlighted both strengths and weaknesses with the game. Nine responses referenced the game being too difficult or confusing. For example, more detail was given around concerns with ease of use: ‘The instructions were long and confusing. The game was a bit hard.’ Six responses also suggested specific changes to the game design or structure.

There were also positive responses; four highlighted how useful the game might be or that it was fun. For example, one participant explained the game is: ‘Fun and easy, good info, fantastic examples, very engaging.’ One suggested the game ‘Really identifies key factors—I think great way to get information to carers. Could be good for patients as well.’

## 6 Discussion

The aim of this study was to develop an SG designed to improve awareness, teach management strategies and reduce the stigma of constipation targeted at those who care for PwID.

A collaborative partnership between Cornwall College, clinical NHS staff and academic staff at the University of Plymouth medical school led to the design of the novel SG ‘poo manager’. The design and development were informed in part by the results of the team's previous literature review and the joint expertise of the partners [44]. Poo manager has been developed to teach carers of PwID how to manage constipation. As challenging gameplay has been proven to improve player motivation, further levels will be developed [55].

As co‐production is vital for the success of any digital intervention [51], potential end users were invited to try the game and provide feedback via a survey to enable further development of the game. The survey identified that the use of non‐pharmacological approaches in preventing and managing constipation is seen as important to our co‐production members, which is supported by the literature that indicates that non‐pharmaceutical options are safer and effective and must be the first step in managing constipation in adults [30, 62]. This supports the design choice in Poo Manager to include several non‐pharmaceutical options within the choices of actions.

The co‐production members were broadly positive about the game's appropriateness, feasibility and acceptability (AIM, IAM and FIM), outside of small improvements required to improve the game's ease of play. These 3 implementation outcome measures are well validated in the literature [60, 61, 63] and have been widely used across health care settings [64, 65, 66, 67]. Therefore, such findings are encouraging, suggesting Poo Manager could be implemented successfully in practice.

Specifically, in terms of acceptability, the results suggest members welcome and like poo manager, but there is a need to develop it further, so it is more appealing and meets higher levels of approval. There were positive results for the appropriateness outcomes, suggesting members find the game has relevance, but the lower score of ‘seems suitable’ (58%) could again be a further indication that the game needs further development. Feasibility outcomes were positive, suggesting the views of members were that poo manager could be successfully implemented in care settings.

There was a much harsher response within the ‘attitudes to poo manager’ section of the survey. Overall, respondents held positive attitudes towards poo manager's entertainment and educational value. However, there were mixed responses to the games perceived effectiveness, likelihood of uptake by care companies and usability, entertainment and education value. This highlights the importance of usability testing an SG before completing all stages of development [68], especially as poor usability testing and game evaluation have been identified as major roadblocks in SG success [69]. Learning from usability testing is vital to effective SG production [70] and the poo manager team will be able to take these issues forward and iron them out in further development.

Even if an improvement in knowledge can be demonstrated, a key challenge for healthcare SGs is translating these into improved patient outcomes. A *poo manager* must bridge this gap to be effective. Recent research has highlighted a recurring issue—many SGs, such as those aimed at enhancing asthma management knowledge, have failed to translate learning gains into measurable clinical improvements [71]. In this evaluation cycle, objective patient outcomes were not assessed; future iterations should incorporate such measures to establish clinical impact.

The literature has demonstrated that it is, however, possible to overcome this issue through a rigorous design process. In a 2020 systematic review of gamification in diabetes care, diabetes SGs, grounded in established educational theory and developed by graphic design experts, successfully translated improvements in knowledge into enhanced patient outcomes [72]. Therefore, Poo Manager could benefit from directly evaluating whether improved knowledge translates into patient outcomes while also implementing theory‐backed design strategies to support this translation.

### 6.1 Limitations

During the co‐production day, game developers worked alongside members of the co‐production workshop. Some of the feedback to the game happened during these interactions and so was not captured in the survey and the results of this paper. Members were encouraged to share their views in the survey, but some of this feedback may not have been captured.

PwID made up 35% (N = 8/23) of the survey group. This is important because, although they are not the end users, it is vital that we gain their understanding based on lived experience. However, they might not have fully appreciated their role in providing advice as experts by experience and may have instead responded as end users. This reflects a weakness in the pre‐survey briefing, and some comments may indicate this.

The version of poo manager used in the co‐production day involved only level one, and so no feedback could be given on the increased difficulty of the game as it goes through the higher levels. Further co‐production work is needed before the game design is finalised to ensure that end user experience is fed back into the design process.

The use of AIM, IAM and FIM to evaluate the response to the intervention is limited by the fact that all of the tool items are positive validation statements. In addition, the survey was designed to examine the perceived efficacy of the intervention, not the pure efficacy itself. Once the poo manager game design process is complete, it will need to be evaluated using a standardised pre/post‐test RCT methodology to see if it is effective.

## 7 Conclusion

The game design team, working collaboratively with patients and end users, successfully developed a novel SG aimed at teaching constipation management to carers of PwID. This is an important initiative, as constipation poses a significant and potentially life‐threatening risk to the morbidity and mortality of PwID.

While the evaluation of poo manager, through a quantitative survey at the co‐production day, demonstrated that there is positive support for the game's acceptability, feasibility and appropriateness (FIM, AIM and IAM), neutral responses in the second ‘attitudes to poo manager’ to elements such as the game's ‘ease of play’, ‘education and entertainment value’ and its likelihood of ‘uptake’ by care companies demonstrate that there are still areas for improvement within the game design.

Further research should focus on improving poo manager's feasibility and game mechanics, by consulting with SG design professionals, and on more reliably evaluating the game's effectiveness. A pre‐post‐test methodology within a randomised control trial to determine how significantly the game improves constipation management knowledge compared to standard didactic teaching methods is the gold standard and would assess whether this is translating into improved patient outcomes.

## Author Contributions

**Serena Daniel**: methodology, data curation, visualisation, investigation, writing – original draft preparation. **Ruth Bishop**: methodology, data curation, visualisation, investigation, validation, writing – original draft preparation. **Stephen Howard**: software, methodology, visualisation, writing – reviewing and editing. **Sarah Lennard**: methodology, visualisation, supervision, validation, writing – reviewing and editing. **Samantha Pye**: project administration, visualisation, writing – reviewing and editing. **Richard Laugharne**: methodology, supervision, visualisation, writing – reviewing and editing. **Christopher James**: visualisation, supervision, validation, writing – reviewing and editing. **Rohit Shankar**: conceptualisation, methodology, data curation, visualisation, supervision, validation, writing – reviewing and editing.

## Funding

The authors have nothing to report.

## Ethics Statement

The NHS Health research authority tool (http://www.hra‐decisiontools.org.uk/research/index.html) confirmed no formal NHS Ethics approval was required. All participants were advised at the start of the study that participation was voluntary and their replies, if they chose to participate, would be anonymised and analysed. Written informed consent was taken from all participants.  No participant identifier data was collected. Data was pooled prior to analysis. Further, it was to a participant group who were participating in co‐production as experts by experience.

## Conflicts of Interest

RS developed the non‐commercial and free to use SUDEP and Seizure Safety Checklist and the EpSMon app to reduce the risk of SUDEP and enhance seizure safety. RS is the chief Investigator of the NIHR adopted national Ep‐ID register. The Register is supported and monitored by the National Institute of Health Research UK. The funding for each molecule examined by the Register is via an Investigator Initiated Support grant from each of the molecule's parent company. The funding is to RS's NHS institution and goes towards the salary of the research coordinator and the institution's project oversight costs. The contributing companies till date include Eisai, UCB, Bial, Jazz pharma (previously GW pharma) and Angelini. This work sits outside the submitted work. In addition to the above RS has received institutional research, travel support and/or honorarium for talks and expert advisory boards from LivaNova, UCB, Eisai, Neuraxpharm, Veriton Pharma, Bial, Angelini, UnEEG and Jazz/GW pharma outside the submitted work. He holds or has held competitive grants from various national grant bodies including Innovate, Economic and Social Research Council (ESRC), Engineering and Physical Sciences Research Council (ESPRC), National Institute of Health Research (NIHR), NHS Small Business Research Initiative (SBRI) and other funding bodies including charities all outside this work. No other author has any declared conflict of interest related to this paper. RL has been Chief Investigator for clinical trials sponsored by Janssen and Boehringer Ingelheim outside the submitted work. There is no direct disclosure or conflict of interest for any author for this submitted body of work.

## Supporting information




**Supplementary File 1**: htl270024‐sup‐0001‐SuppMat.jpg.

**Supplementary File 2**: htl270024‐sup‐0002‐SuppMat.jpg.

**Supplementary File 3**: htl270024‐sup‐0003‐SuppMat.jpg.

**Supplementary File 4**: htl270024‐sup‐0004‐SuppMat.jpg.

**Supplementary File 5**: htl270024‐sup‐0005‐SuppMat.jpg.

**Supplementary File 6**: htl270024‐sup‐0006‐SuppMat.jpg.

**Supplementary File 7**: htl270024‐sup‐0007‐SuppMat.jpg.

**Supplementary File 8**: htl270024‐sup‐0008‐SuppMat.jpg.

**Supplementary File 9**: htl270024‐sup‐0009‐SuppMat.jpg.

**Supplementary File 10**: htl270024‐sup‐0010‐SuppMat.jpg.

**Supplementary File 11**: htl270024‐sup‐0011‐SuppMat.jpg.

**Supplementary File 12**: htl270024‐sup‐0012‐SuppMat.jpg.

**Supplementary File 13**: htl270024‐sup‐0013‐SuppMat.jpg.

**Supplementary File 14**: htl270024‐sup‐0014‐SuppMat.jpg.

**Supplementary File 15**: htl270024‐sup‐0015‐SuppMat.jpg.

**Supplementary File 16**: htl270024‐sup‐0016‐SuppMat.docx.

## Data Availability

All data used for the paper is within the manuscript.
