# Supplementary figures and images for: Poo Manager: Co‐Designing a Serious Computer Game to Improve Constipation Management Awareness in Carers of People With Intellectual Disabilities
Source: Healthc Technol Lett. 2025 Nov 3;12(1):e70024. doi: 10.1049/htl2.70024 (PMC12583887; doi:10.1049/htl2.70024)

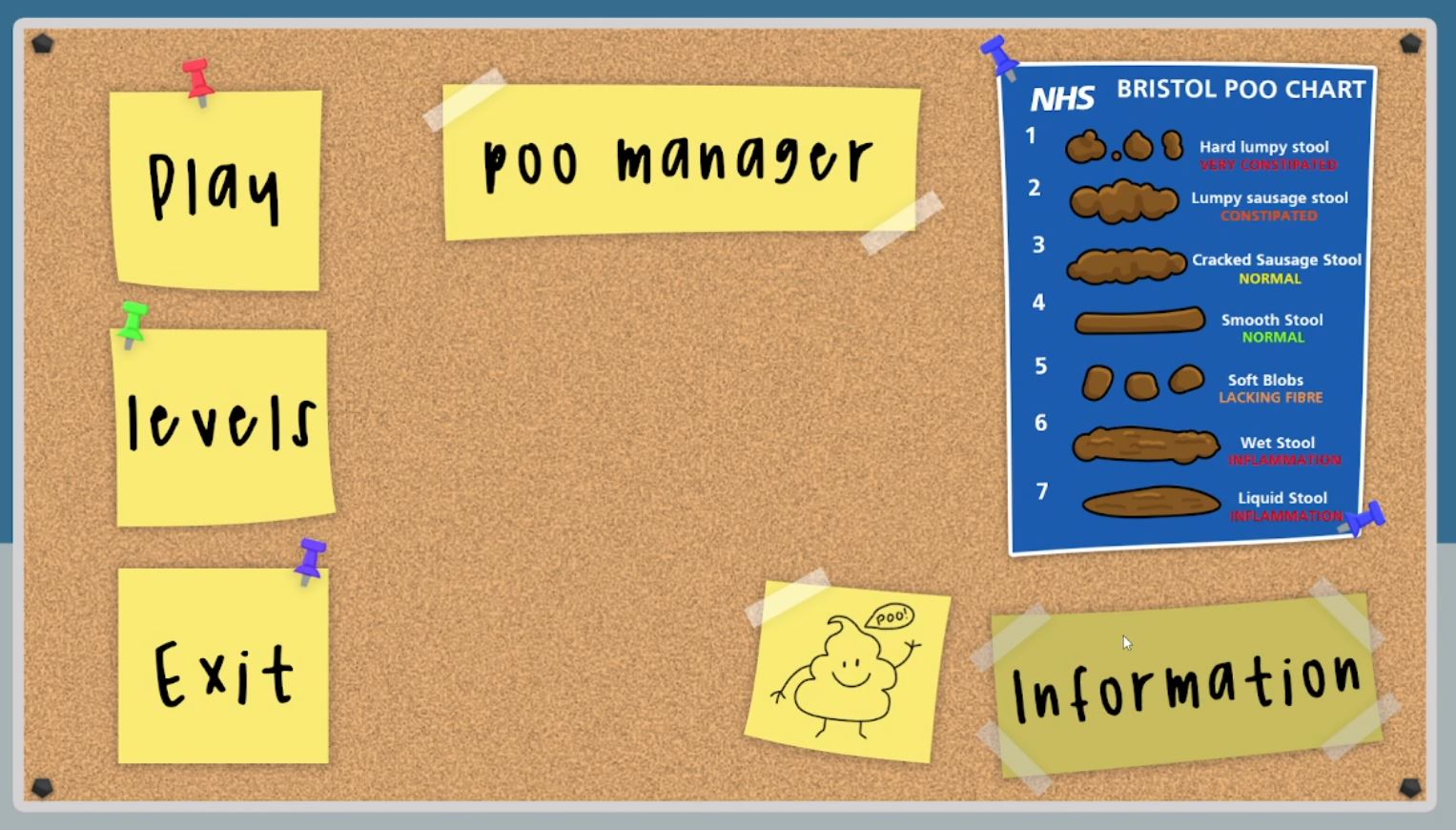

Supplement: Supplementary file 1 — htl270024‐sup‐0001‐SuppMat.jpg. [file HTL2-12-e70024-s004.jpg]

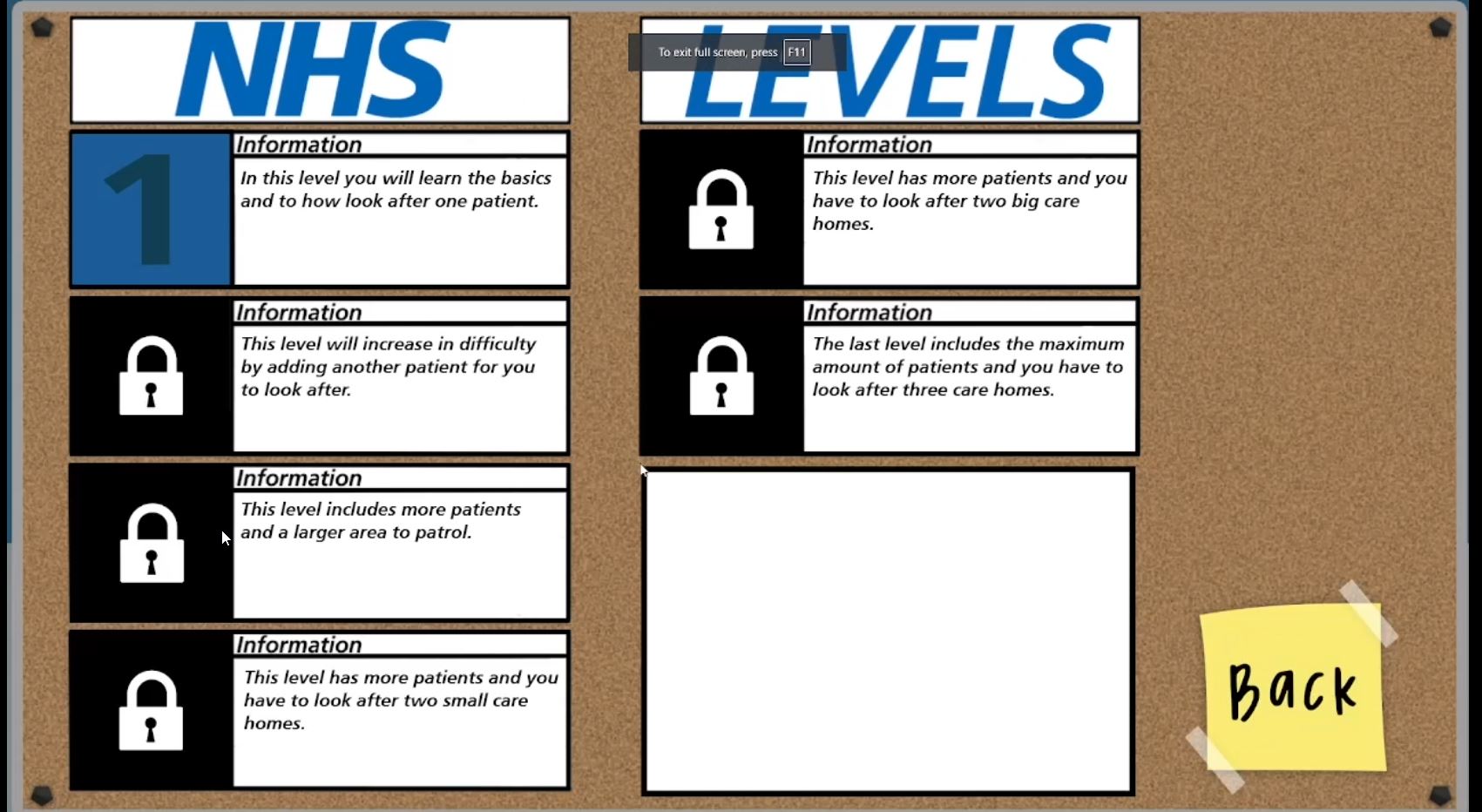

Supplement: Supplementary file 2 — htl270024‐sup‐0002‐SuppMat.jpg. [file HTL2-12-e70024-s013.jpg]

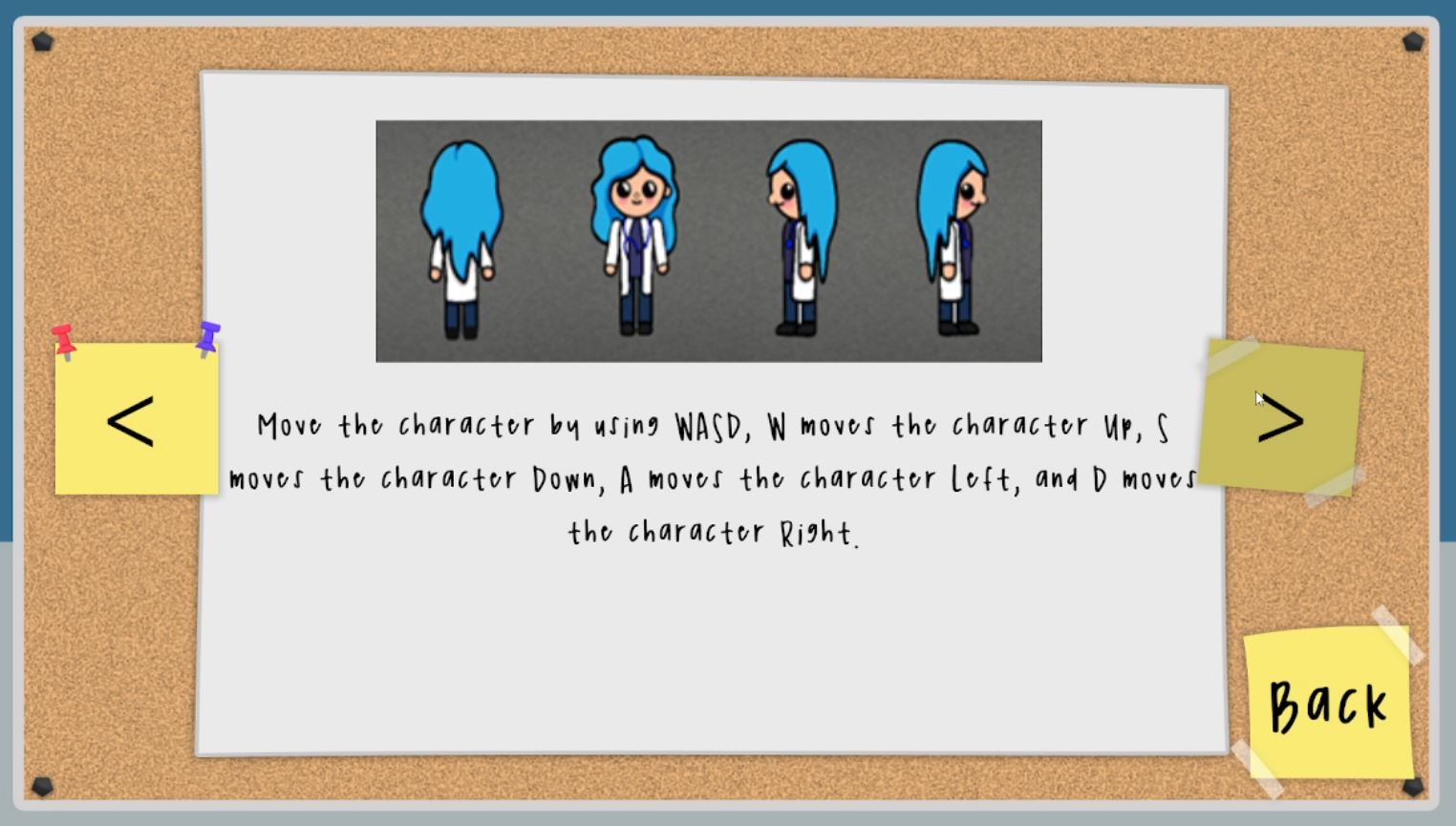

Supplement: Supplementary file 3 — htl270024‐sup‐0003‐SuppMat.jpg. [file HTL2-12-e70024-s006.jpg]

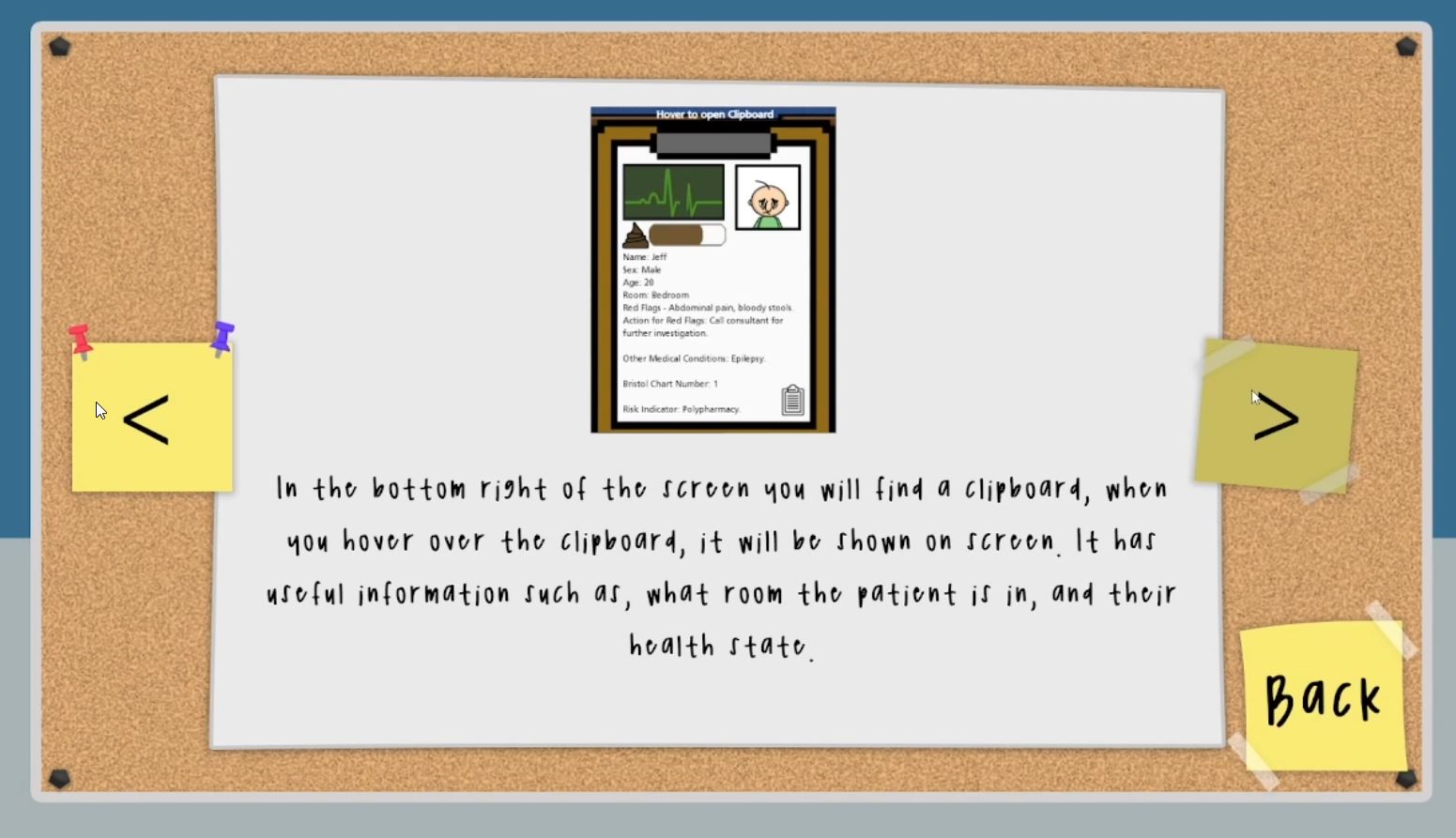

Supplement: Supplementary file 4 — htl270024‐sup‐0004‐SuppMat.jpg. [file HTL2-12-e70024-s003.jpg]

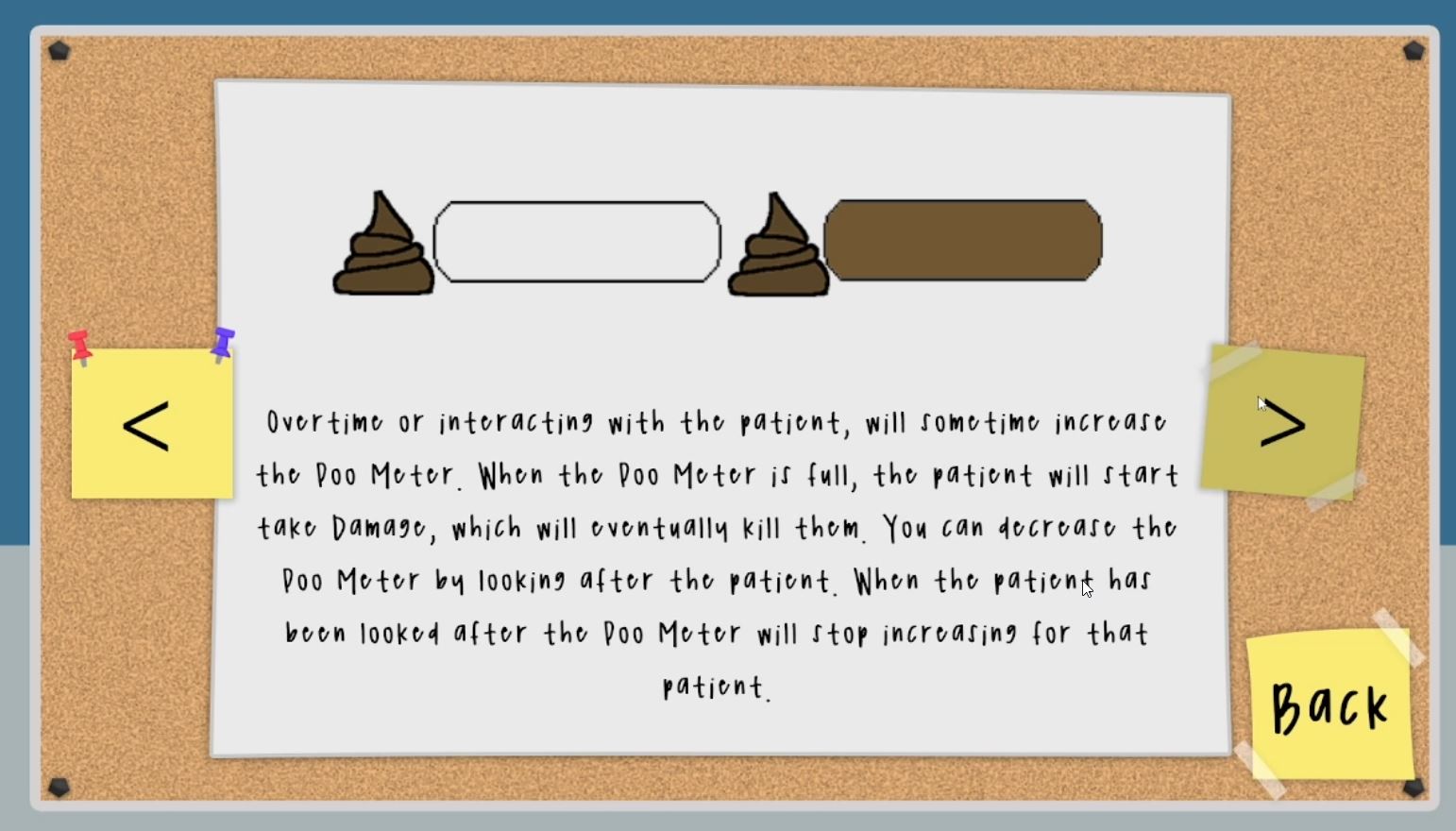

Supplement: Supplementary file 5 — htl270024‐sup‐0005‐SuppMat.jpg. [file HTL2-12-e70024-s001.jpg]

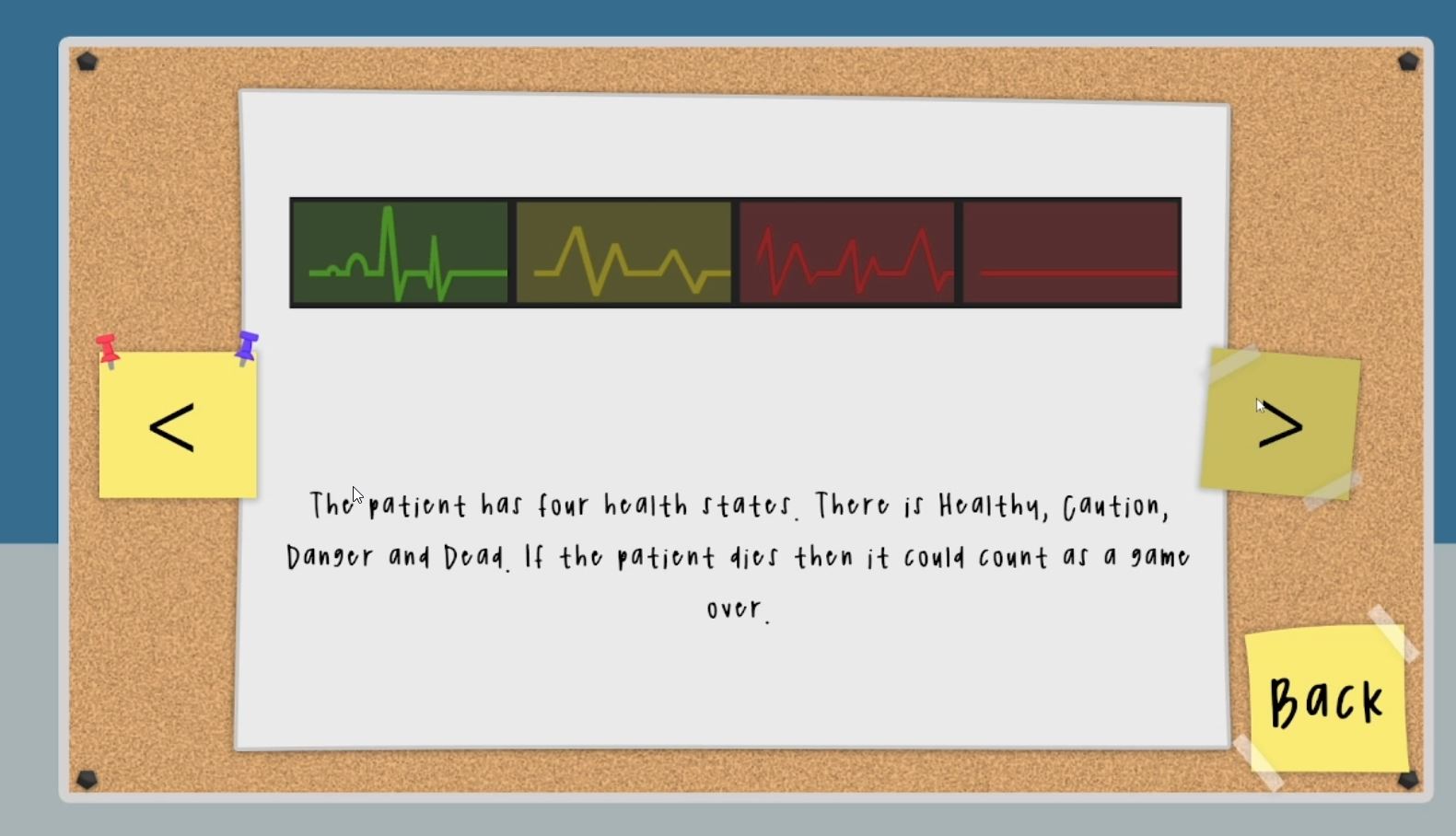

Supplement: Supplementary file 6 — htl270024‐sup‐0006‐SuppMat.jpg. [file HTL2-12-e70024-s005.jpg]

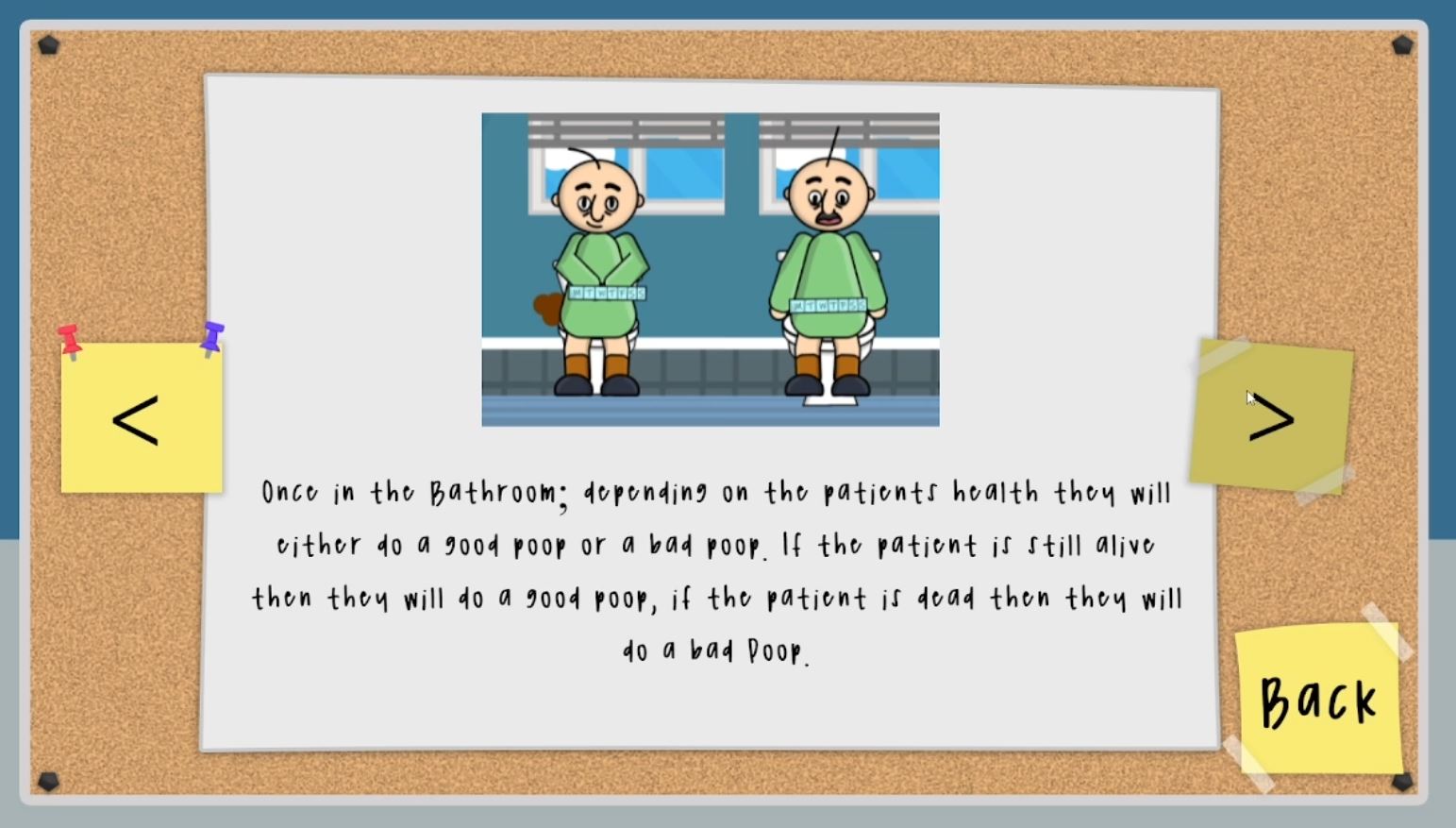

Supplement: Supplementary file 7 — htl270024‐sup‐0007‐SuppMat.jpg. [file HTL2-12-e70024-s015.jpg]

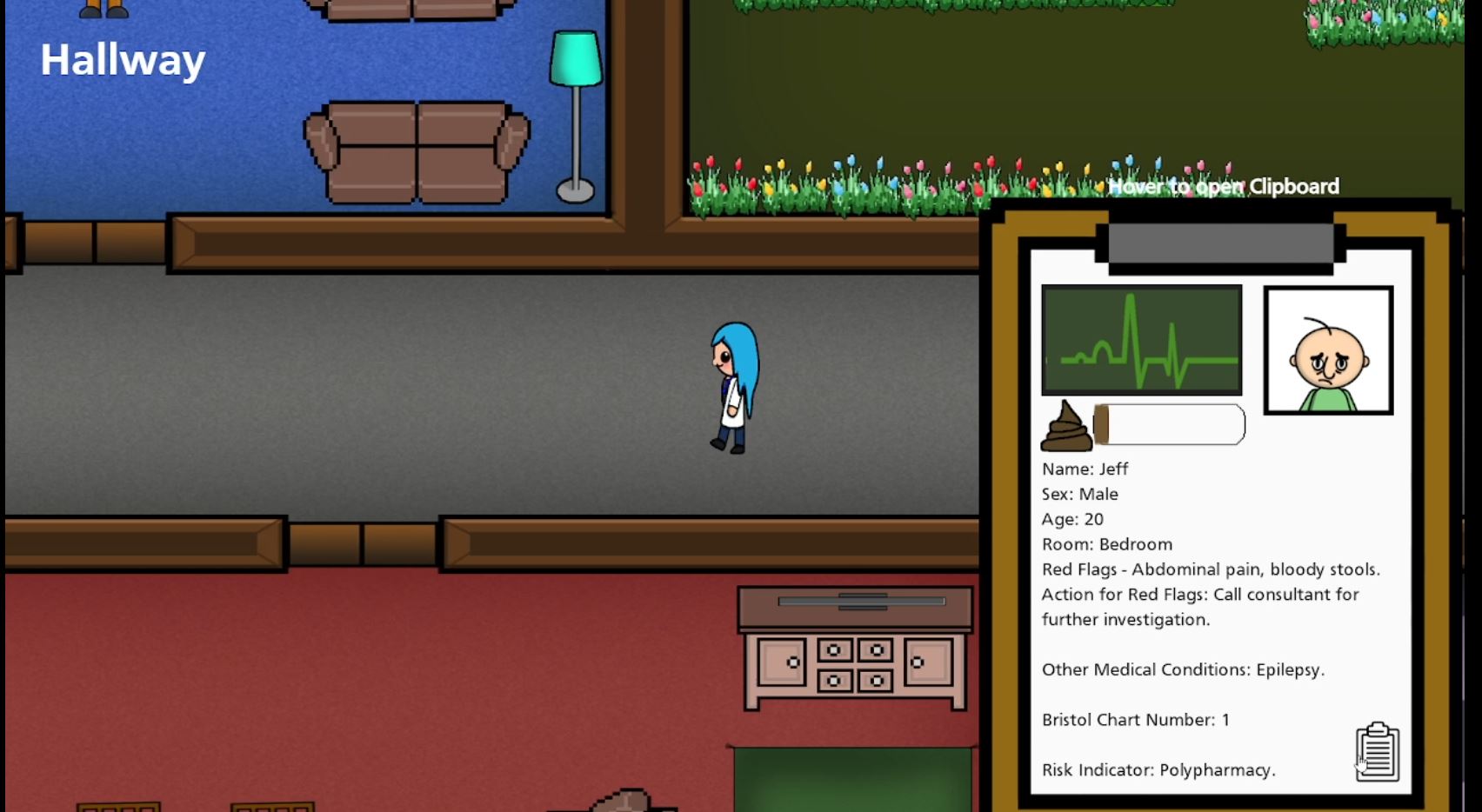

Supplement: Supplementary file 8 — htl270024‐sup‐0008‐SuppMat.jpg. [file HTL2-12-e70024-s014.jpg]

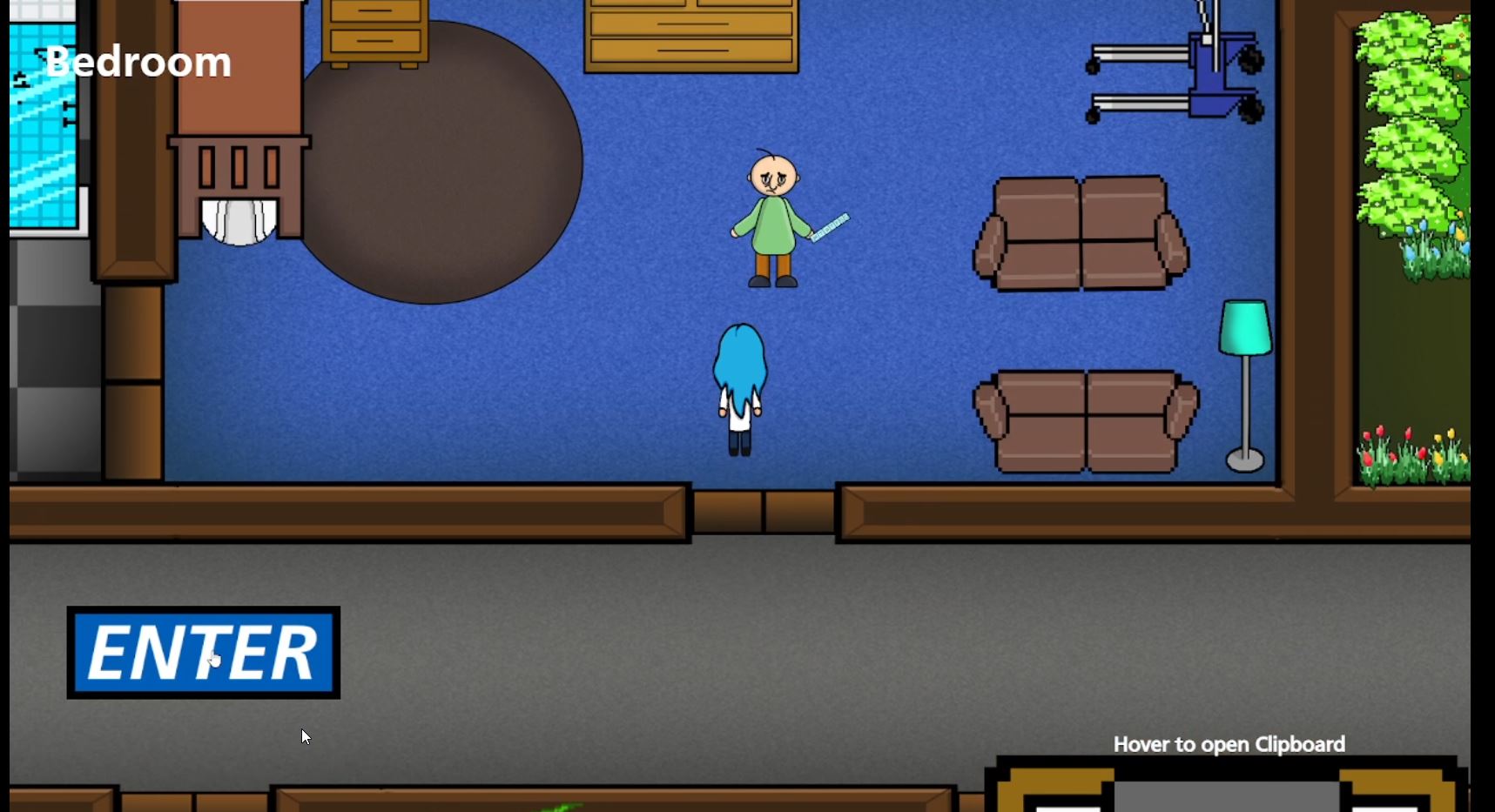

Supplement: Supplementary file 9 — htl270024‐sup‐0009‐SuppMat.jpg. [file HTL2-12-e70024-s009.jpg]

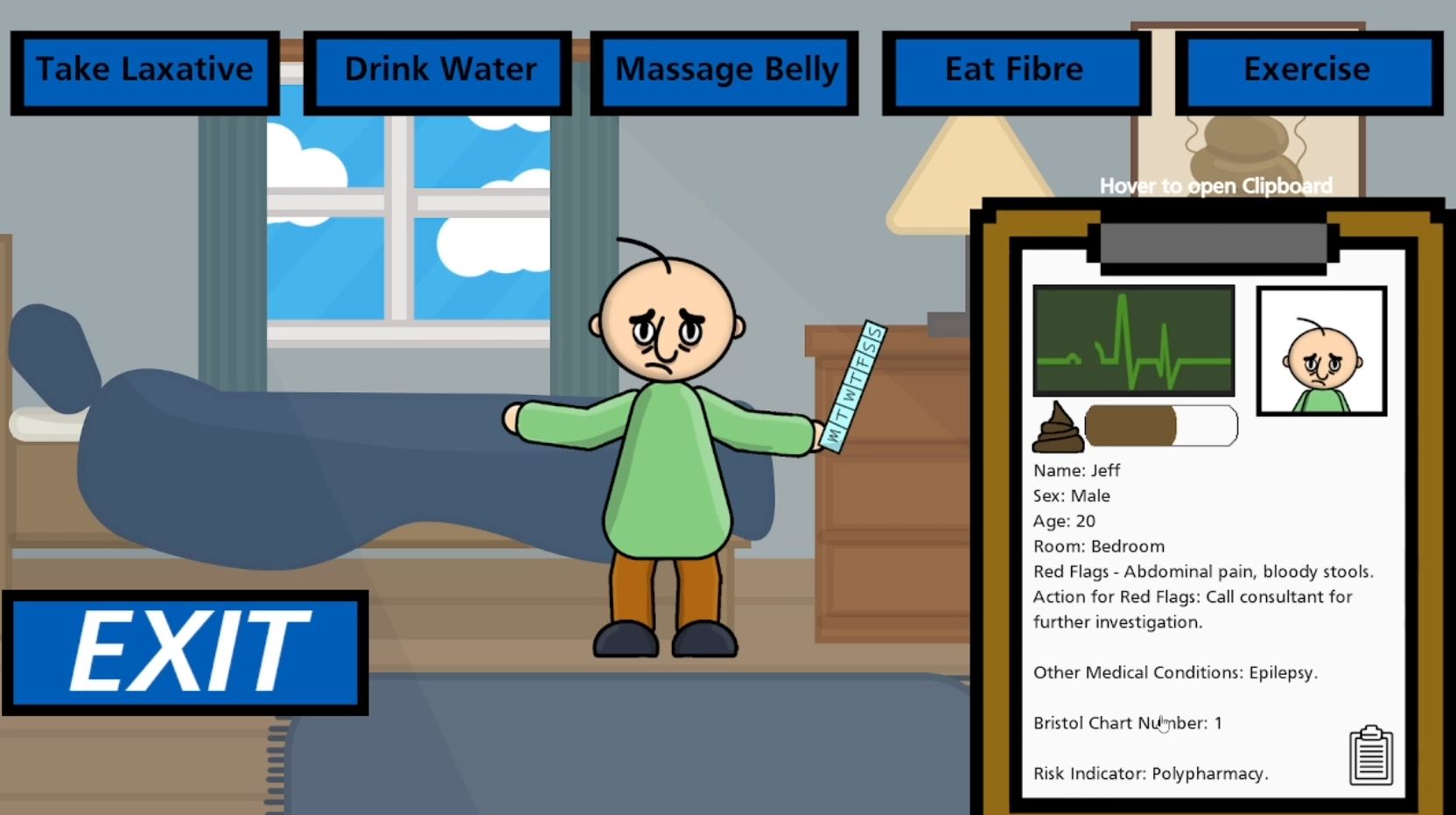

Supplement: Supplementary file 10 — htl270024‐sup‐0010‐SuppMat.jpg. [file HTL2-12-e70024-s008.jpg]

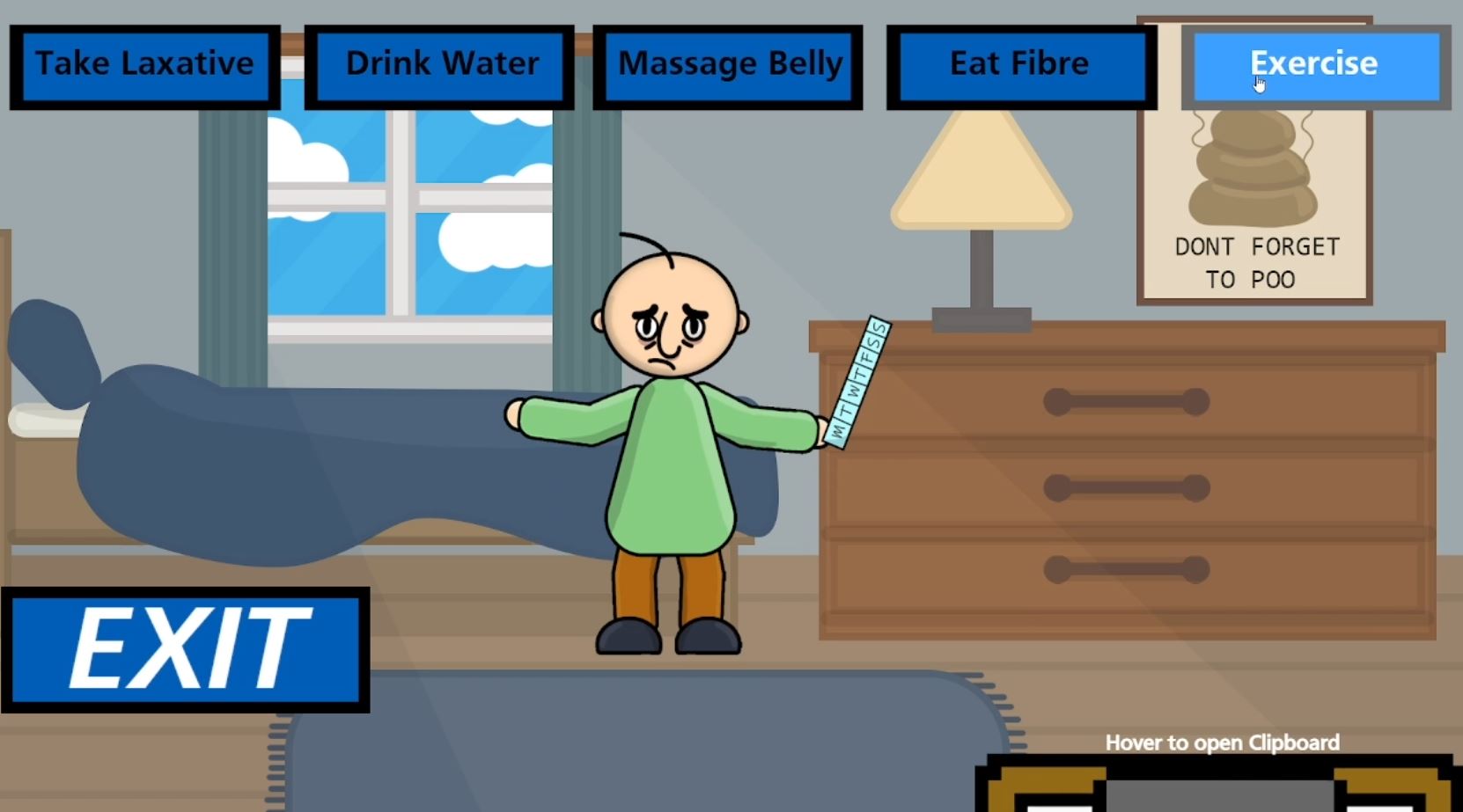

Supplement: Supplementary file 11 — htl270024‐sup‐0011‐SuppMat.jpg. [file HTL2-12-e70024-s010.jpg]

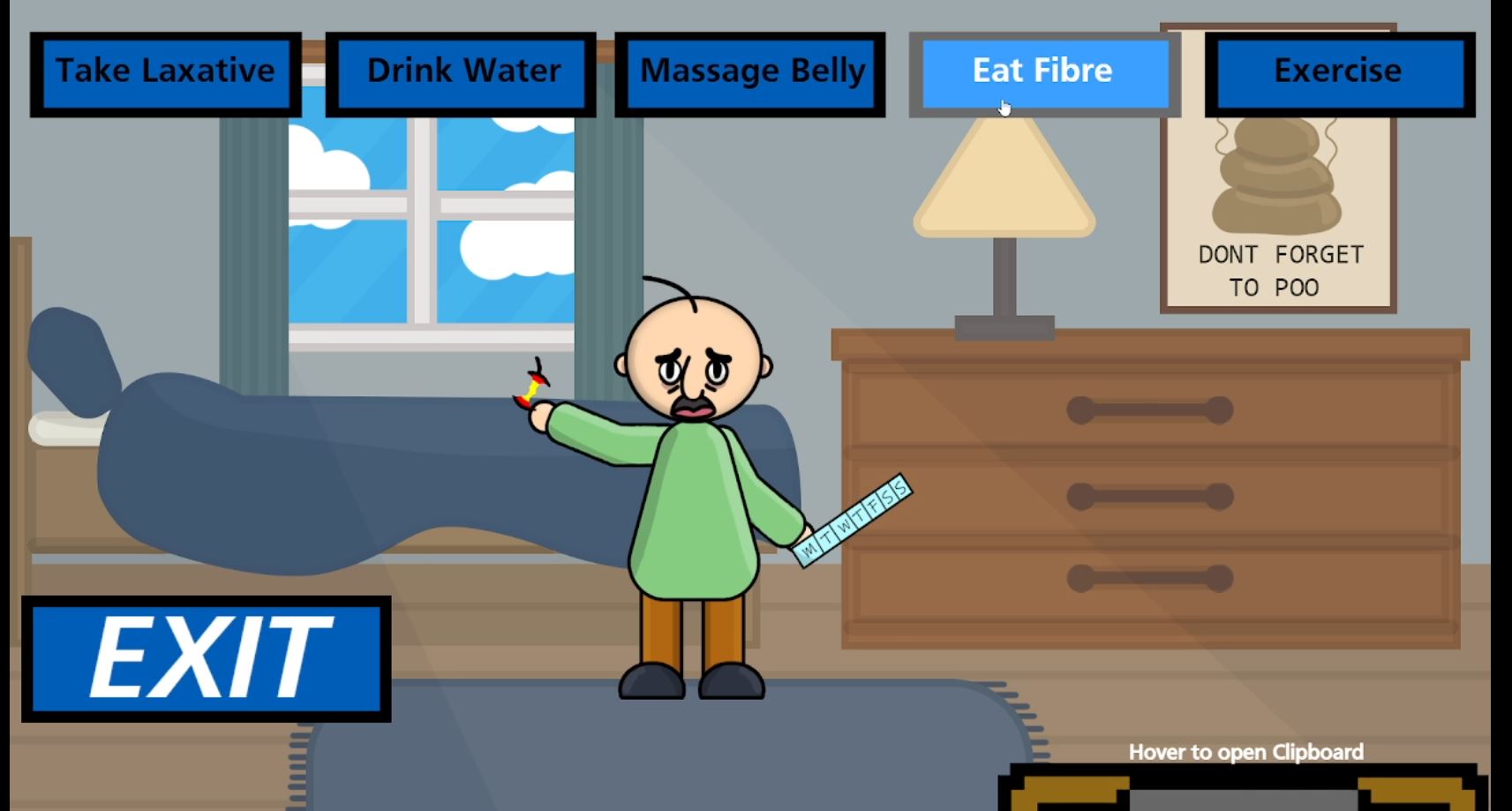

Supplement: Supplementary file 12 — htl270024‐sup‐0012‐SuppMat.jpg. [file HTL2-12-e70024-s002.jpg]

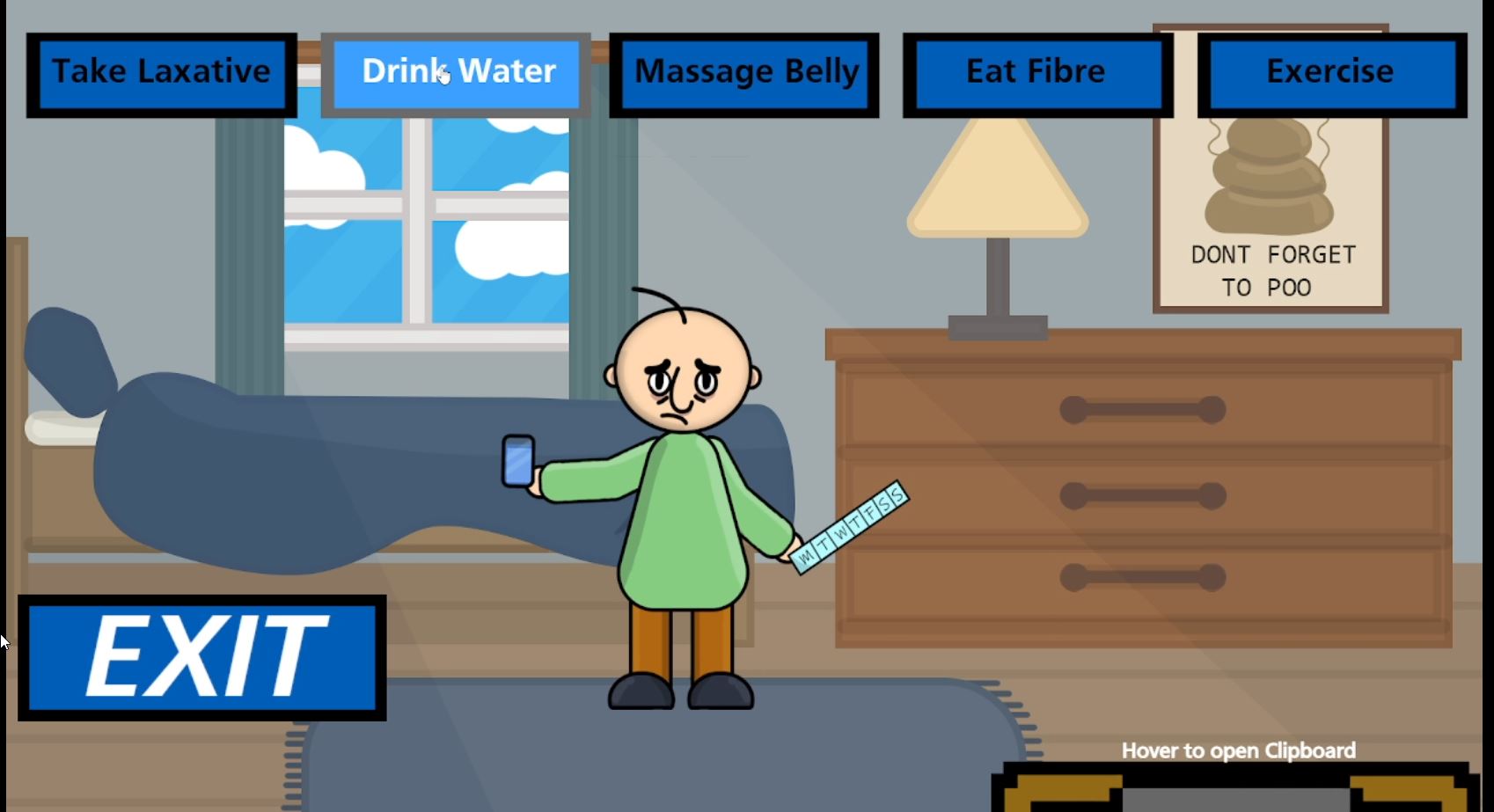

Supplement: Supplementary file 13 — htl270024‐sup‐0013‐SuppMat.jpg. [file HTL2-12-e70024-s011.jpg]

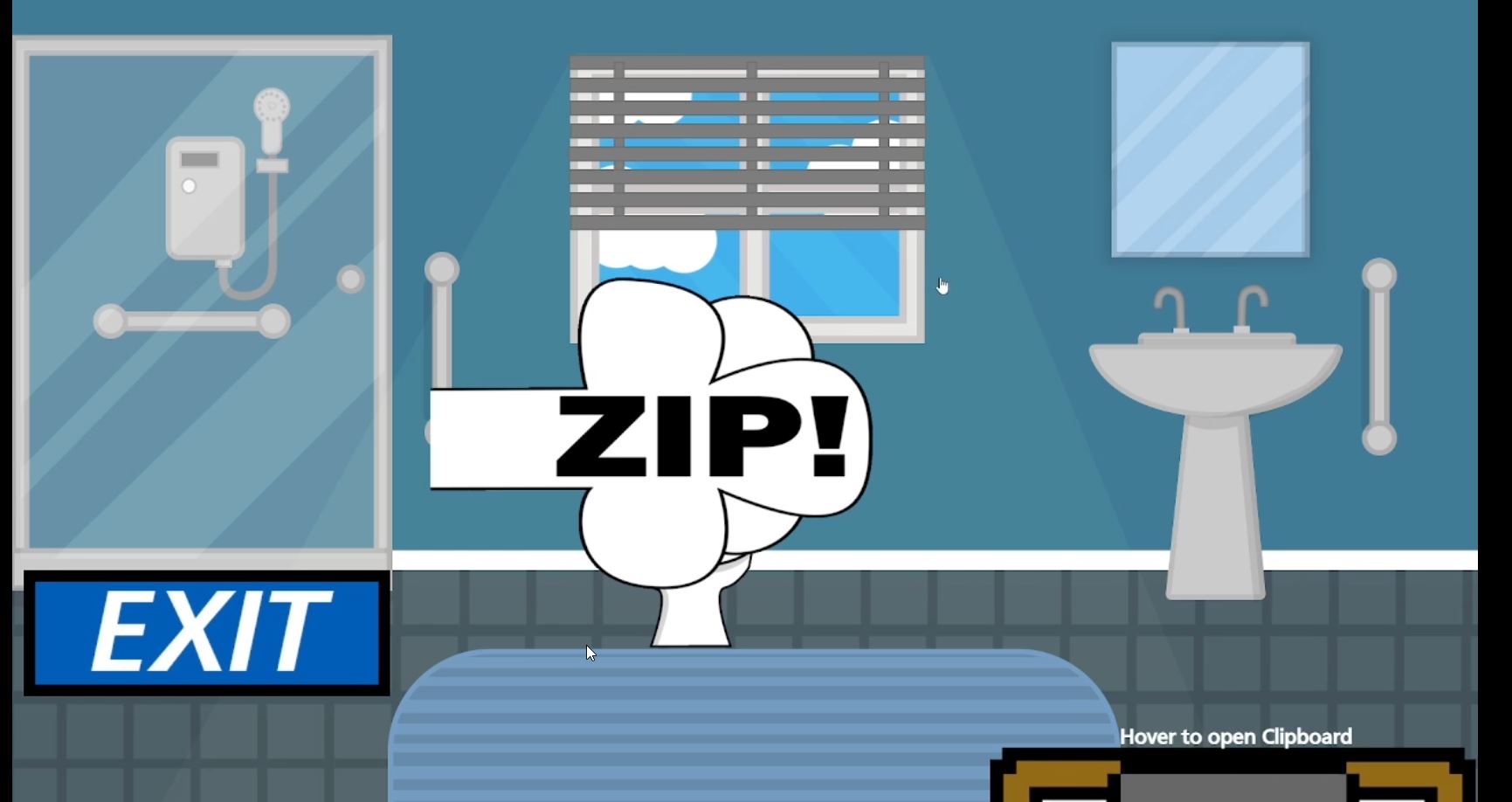

Supplement: Supplementary file 14 — htl270024‐sup‐0014‐SuppMat.jpg. [file HTL2-12-e70024-s012.jpg]

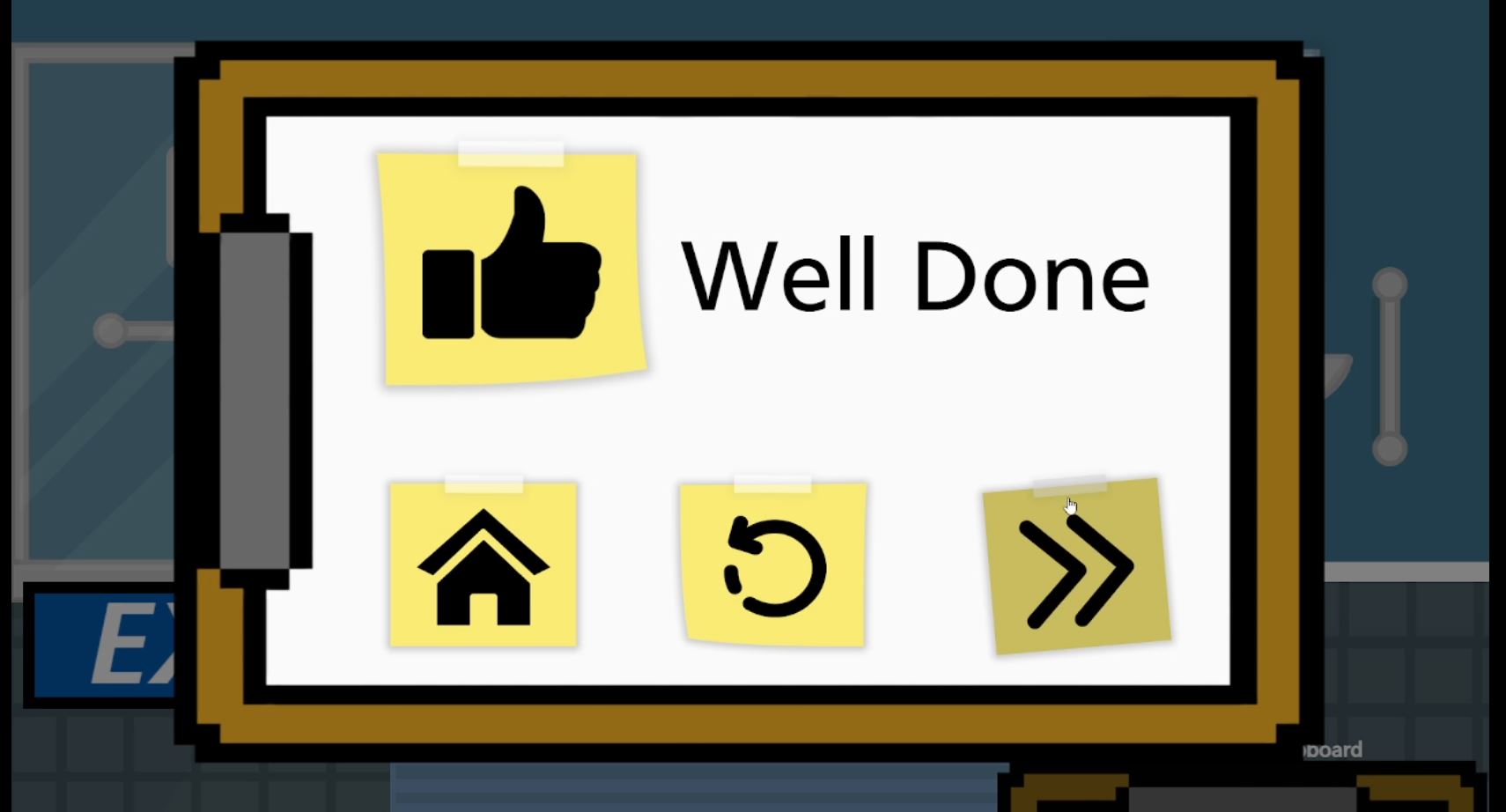

Supplement: Supplementary file 15 — htl270024‐sup‐0015‐SuppMat.jpg. [file HTL2-12-e70024-s007.jpg]
