# Supplementary material for: Poo Manager: Co‐Designing a Serious Computer Game to Improve Constipation Management Awareness in Carers of People With Intellectual Disabilities
Source: Healthc Technol Lett. 2025 Nov 3;12(1):e70024. doi: 10.1049/htl2.70024 (PMC12583887; doi:10.1049/htl2.70024)
Supplement: Supplementary file 16 — htl270024‐sup‐0016‐SuppMat.docx. [file HTL2-12-e70024-s016.docx]

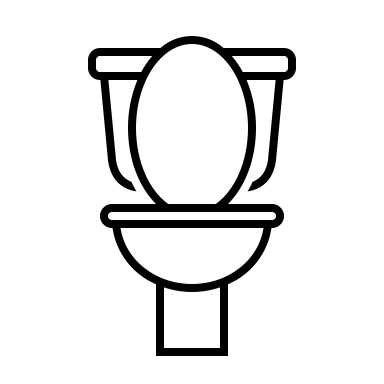
**Poo Manager Feedback Questionnaire**
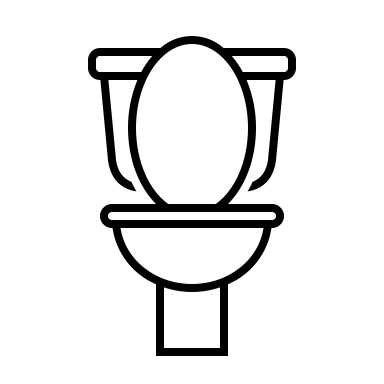


*Please ask a member of the team if you would like help to complete the form.*

- *I understand that the feedback I give may be used anonymously in future research publications.*

*(Please put your initials in the box to*

*confirm you understand and agree)*

- *All information will be collected anonymously. All personal information will remain confidential.*
- *The data from the questionnaire will be stored securely.*
- *If you have any questions, please discuss with a member of staff.*
- *Your participation in the questionnaire is voluntary and you can stop at any time.*

**About you**

1. Gender:…………………………………….
2. Age:…………………………………………..
3. Do you have a learning disability? Yes /No/Not sure
4. Do you support people with a learning disability

as part of your work? Yes/No/Not sure

1. Do you have a family member with

a learning disability? Yes/No/Not sure

1. Have you experienced constipation personally? Yes/No/Not sure
2. Have you supported someone with constipation? Yes/No/Not sure

**About constipation management**

1. Please answer the following statements on a scale of 1-5, with 1 indicating very unimportant, and 5 indicating very important.

|  | **1**  **(very unimportant)** | **2 (unimportant)** | **3 (neutral)** | **4 (important)** | **5**  **(very important)** |
| --- | --- | --- | --- | --- | --- |
| How important is training in constipation management? |  |  |  |  |  |
| How important is the use of medication in constipation management? |  |  |  |  |  |
| How important are non-pharmaceutical methods in constipation management? (e.g. diet, fluid intake) |  |  |  |  |  |
| How important is knowledge of the harm constipation can lead to? |  |  |  |  |  |

**About the game ‘Poo Manager’**

1. Please answer the following statements in relation to the game ‘Poo Manager’ on a scale of 1-5, with 1 indicating strongly disagree and 5 indicating strongly agree.

|  | **1 (strongly disagree)** | **2 (disagree)** | **3 (neither agree or disagree)** | **4 (agree)** | **4 (strongly agree)** |
| --- | --- | --- | --- | --- | --- |
| The game is easy to use. |  |  |  |  |  |
| The game is fun to play. |  |  |  |  |  |
| The game will help carers learn about how to manage constipation. |  |  |  |  |  |
| Carers and care companies will use the game. |  |  |  |  |  |
| The game will help reduce constipation. |  |  |  |  |  |
| The game will help carers learn about different ways to prevent and treat constipation. |  |  |  |  |  |

Please use the space below to write any other thoughts or comments.

…………………………………………………………………………………………………………………………………………………………………………………………………………………………………………………………………………………………………………………………………………………………………………………………………………………………………………………………………………………………………………………………………………………………………………………………………………………………………………………………………………………………………………………………………………………………………………………………………………………………………………………………………………………………

*Thank you!*

**Additional questions on implementation**

**Acceptability of Intervention Measure (AIM)**

|  | Completely disagree | Disagree | Neither agree nor disagree | Agree | Completely agree |
| --- | --- | --- | --- | --- | --- |
| 1. ‘Poo Manager’ meets my approval. | ➀ | ➁ | ➂ | ➃ | ➄ |
| 2. ‘Poo Manager’ is appealing to me. | ➀ | ➁ | ➂ | ➃ | ➄ |
| 3. I like ‘Poo Manager’. | ➀ | ➁ | ➂ | ➃ | ➄ |
| 4. I welcome ‘Poo Manager. | ➀ | ➁ | ➂ | ➃ | ➄ |

**Intervention Appropriateness Measure (IAM)**

|  | Completely disagree | Disagree | Neither agree nor disagree | Agree | Completely agree |
| --- | --- | --- | --- | --- | --- |
| 1. ‘Poo Manager’ seems fitting. | ➀ | ➁ | ➂ | ➃ | ➄ |
| 2. ‘Poo Manager’ seems suitable. | ➀ | ➁ | ➂ | ➃ | ➄ |
| 3. ‘Poo Manager’ seems applicable. | ➀ | ➁ | ➂ | ➃ | ➄ |
| 4. ‘Poo Manager’ seems like a good match. | ➀ | ➁ | ➂ | ➃ | ➄ |

**Feasibility of Intervention Measure (FIM)**

|  | Completely disagree | Disagree | Neither agree nor disagree | Agree | Completely agree |
| --- | --- | --- | --- | --- | --- |
| 1. ‘Poo Manager’ seems implementable. | ➀ | ➁ | ➂ | ➃ | ➄ |
| 2. ‘Poo Manager’ seems possible. | ➀ | ➁ | ➂ | ➃ | ➄ |
| 3. ‘Poo Manager’ seems doable. | ➀ | ➁ | ➂ | ➃ | ➄ |
| 4. ‘Poo Manager’ seems easy to use. | ➀ | ➁ | ➂ | ➃ | ➄ |
